# Tube2FEM: a general-purpose highly automated pipeline for flow-related processes in (embedded) tubular objects

**DOI:** 10.1098/rsos.242025

**Published:** 2025-08-13

**Authors:** Hani Cheikh Sleiman, Kevin M. Moerman, Diana C. De Oliveira, Joseph Jacob, Nesrin Mogulkoc, Brian R. Davidson, Simon Walker-Samuel, Rebecca J. Shipley

**Affiliations:** ^1^ Department of Mechanical Engineering, University College London, London, UK; ^2^ Centre for Computational Medicine, University College London, London, UK; ^3^ Department of Mechanical Engineering, University of Galway, Galway, Ireland; ^4^ Satsuma Lab, Hawkes Institute, University College London, London, UK; ^5^ UCL Respiratory, University College London, London, UK; ^6^ Department of Respiratory Medicine, Ege University Hospital, Izmir, Turkey; ^7^ Wellcome/EPSRC Centre for Interventional and Surgical Sciences (WEISS), University College London, London, UK; ^8^ Centre for Surgical Innovation, Organ Repair and Transplantation (CSIORT), University College London, London, UK; ^9^ Royal Free Hospital NHS Trust, London, UK; ^10^ Centre for Advanced Biomedical Imaging, University College London, London, UK

**Keywords:** computational fluid dynamics, 3D–1D mixed-dimension, embedded networks, vascular networks, conforming mesh, lung airways

## Abstract

This paper presents an open-source pipeline for simulating flow and flow-related processes in (embedded) tubular structures. Addressing a gap in computational fluid dynamics (CFD) and simulation sciences, it facilitates the transition from raw three-dimensional imaging, graph networks or computer aided design (CAD) models of tubular objects to refined, simulation-ready meshes. This transition, traditionally labour-intensive, is streamlined through a series of innovative steps that include surface mesh processing, centre-line construction, anisotropic mesh generation and volumetric meshing, leading to finite element method (FEM) simulations. The pipeline leverages a range of open-source software and libraries, notably *GIBBON*, *FEniCS* and *Paraview*, to provide flexibility and broad applicability across different simulation scenarios, ranging from biomedical to industrial applications. We demonstrate the versatility of our approach through five applications, including the mesh generation for soil–root systems, lung airways, microcirculation networks and portal vein networks, each originating from a different data source. Moreover, for several of these cases, we incorporate CFD simulations and strategies for 3D–1D coupling between the embedding domain and the embedded structures. Finally, we outline some future perspectives aimed at enhancing accuracy, reducing computational time and incorporating advanced modelling and boundary condition strategies to further refine the framework’s capabilities.

## 1. Introduction

In the rapidly evolving field of computational fluid dynamics (CFD) and simulation sciences, the precise and accurate representation of tubular structures, including those embedded within complex environments, emerges as a crucial element across a broad spectrum of applications. These applications span diverse fields, from detailed biophysical interactions within root–soil systems, as evidenced by previous studies [1–6], to the exploration and biomimicry of ant nest architectures and their tunnelling strategies [7,8]. They extend further to the biomedical realm from the modelling of flows in blood vessels [9–11] and within lung airways [12,13] to understanding tissue perfusion dynamics [14–20]. The relevance of (embedded) tubular structures also extends to industrial applications, for example the design and optimization of heat exchangers [21], the efficiency of oil extraction processes at the scale of wells and reservoirs [22,23] and safety considerations in tunnel ventilation systems [24] through to the engineering complexity involved in ensuring the stability and integrity of pipeline-soil systems [25,26].

Recent developments in imaging techniques (such as synchrotron or laboratory X-ray computed tomography (CT) [27–29], neutron CT [30] and multifluorescence high-resolution episcopic microscopy [31]), coupled with novel segmentation and image processing methods [32,33], have provided unprecedented access to realistic structures comprising tubular objects down to the finest length scales. Alternatively, when the required imaging resolution is lacking, it is possible to algorithmically generate biomimetic and morphologically realistic synthetic tubular objects algorithmically [34–36]. This availability of structural data from imaging or synthetic sources presents both challenges and opportunities. Combining such data with mathematical models of physical processes provides the opportunity to interrogate the link between structural and functional relationships [37]. However, this relies on robust tools to extract the structural features from the raw imaging data [33].

Despite this opportunity, the pathway from obtaining three-dimensional imaging, synthetic data or computer-aided design (CAD) models of (embedded) tubular objects to generating meshes that are ready for numerical simulations presents significant hurdles. This process is often labour-intensive and fraught with challenges, particularly when it requires the transition from raw data to refined, simulation-ready formats. Additionally, when these models are combined with FEM to perform relevant simulations such as CFD and advection–diffusion–reaction simulations, followed by the need for careful post-processing, the whole process becomes much more complex. This issue becomes even more challenging when trying to complete all these steps within a single open-source platform. In fact, the absence of seamless integration between various stages can make workflows more complex, particularly because of the format incompatibilities among different tools. Moreover, implementing an open-source platform often comes at the cost of using different libraries/software and ensuring input/output compatibility. However, open-source platforms increase the transparency and reproducibility of the research and, thereby, accelerate innovation within the scientific community.

Consequently, it is crucial to develop a comprehensive highly automated open-source pipeline that integrates the various steps of the process into a single framework. This unified approach would streamline workflows and promote the use of advanced simulation techniques across a wide range of applications. Such a workflow would mitigate the labour-intensive work associated with processing geometries and enabling the automated analysis of large datasets. This is particularly pivotal in fields such as the biomedical sector, where the capability to efficiently handle pre-clinical data (e.g. from animal models) and/or patient-specific data can significantly enhance diagnostic and therapeutic strategies on large multi-object datasets.

This paper is structured as follows: §2 describes our methods, starting with a comprehensive review of the software choices that underpin our pipeline and highlighting the interoperability of software packages and the automation strategies employed to streamline these processes. The different compartments are then described including the surface mesh processing from network-based and image-based inputs, the skeletonization of tubular structures, the boundary condition automation, the mesh generation and conversion, the FEM for the mathematical models integrated in the framework and finally the post-processing and visualization of the simulation output. We showcase various examples in §3 to demonstrate the applicability and efficacy of our framework across a range of scenarios, underscoring its potential to significantly advance the field of (embedded) tubular structure simulations. Finally, a list of potential enhancements is outlined in §4, delineating the path for future advancements before concluding the paper in §5.

## 2. Methods

### 2.1. Software/package choices

While other open-source frameworks such as *SimVascular* [9] and *CRIMSON* [38] facilitate physics-based simulations of tubular structures, they are primarily tailored for cardiovascular applications and have limited applicability in scenarios involving different physical processes, such as root–soil interactions or embedded tubular systems. In developing our pipeline, we have strategically selected a combination of powerful general-purpose open-source software packages to ensure efficiency and flexibility across various stages of our workflow. It is worth mentioning that all the software and packages were invoked through a unified *MATLAB* script to maximize automation capabilities.

The first part of the pipeline generates and processes surface meshes from different inputs (three-dimensional imaging, CAD models, synthetic/skeletonized networks), using an open-source *MATLAB*-based [39] toolbox called *GIBBON* (the Geometry and Image-Based Bioengineering add-ON) [40]. *GIBBON* includes a wide range of CAD, geometry and image processing tools. This toolbox also enables interfacing with free open-source software such as *TetGen* [41] and *Geogram* [42] that both possess high-quality meshing capabilities.

The second part of our pipeline involves extracting the centre-lines of the tubular structures. We integrate two versatile skeletonization algorithms, *VesselVio* [43] and *TreeSkel* [44], that have been used respectively on vascular and airways datasets.

For the automatic labelling of surface and volumetric mesh generation and conversion in the third and fourth segments of the pipeline, we rely on the features offered by *GIBBON* and *meshio* [45,46]. The *meshio* Python library is designed for handling mesh data. It provides functionalities for reading, writing and converting mesh files in various formats commonly used in computational simulations and visualization.

There are numerous open-source finite element software options which have been developed to solve free fluid flow and Darcy-scale porous-medium flow problems as well as coupled partial differential equation (PDE)-based transport models, such as *$Dumu^{x}$* [47,48] and *FEBio* [49,50] (a detailed list can be found in [51]). We opt for *FEniCS* [52–54], which is a general-purpose open-source software framework designed to streamline the process of discretizing PDE using the FEM. *FEniCS* leverages the capabilities of the unified form language (UFL) [55] and the *FEniCS* form compiler (FFC) [56] to automatically produce optimized low-level C++ code for evaluating equations expressed in finite element variational forms. Additionally, it provides interfaces to PETSc and Trilinos for linear algebra computations. To use *FEniCS*, the user is required to provide the high-level variational form of the differential equation and does not have to perform any coding for the discretized form at the level of the cell and element scale. *FEniCS* is also well-suited for advanced solid mechanics simulations, particularly when integrated with *MFront* [57–59], allowing therefore a coupling between flow and mechanics.

Finally, for post-processing purposes, *ParaView* [60,61] (built on the visualization toolkit (VTK) library [62]) stands out as a comprehensive visualization tool, facilitating intuitive exploration and analysis of simulation outcomes. Its broad array of features and compatibility with multiple data formats render it an essential resource for extracting valuable information from simulation results.

**Figure 1.**
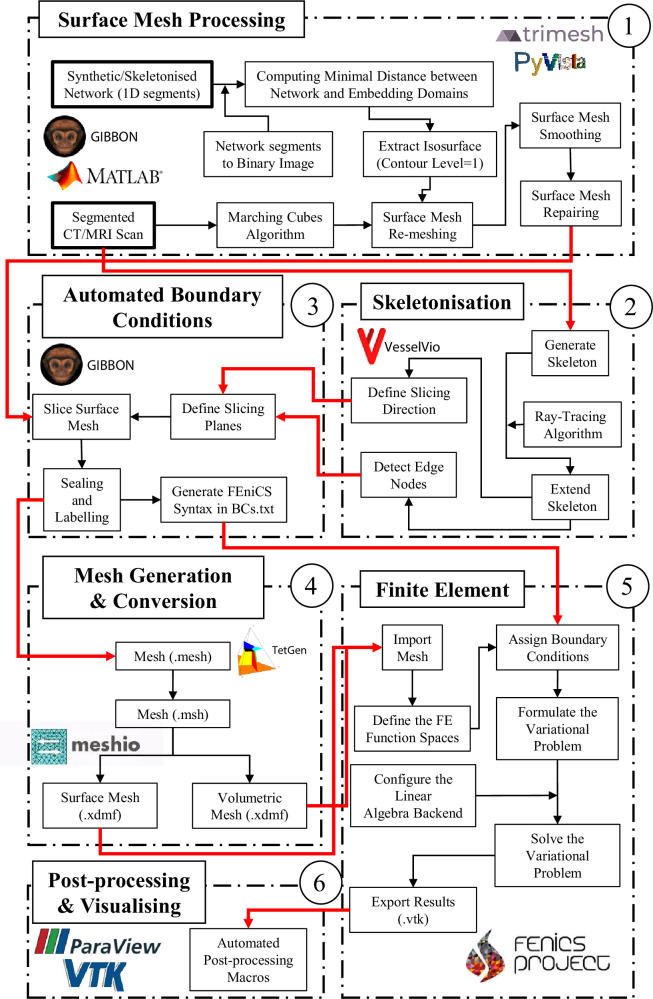
Interoperability of the software/packages used in the Tube2FEM pipeline. Input data is highlighted with a bold contour, inter-module and intra-module operations are represented by red arrows and black arrows, respectively.

Figure 1 illustrates the interaction of the different software/packages used in the Tube2FEM pipeline. Next, we describe the components of this workflow in turn.

### 2.2. Generating a watertight surface mesh

This workflow is intentionally flexible and can handle different inputs, including segmented three-dimensional images issued from different imaging tools (X-ray computed tomography (XTC), optical coherence tomography (OCT), magnetic resonance imaging (MRI), etc.) and synthetic/skeletonized graph networks.

For the first type of input, namely, the segmented three-dimensional image, the creation of the surface mesh can be efficiently accomplished by employing the computer graphics marching cubes algorithm [63]. We use the implementation of the Marching Cube from the *MATLAB* file exchange [64] for this purpose. It is also possible to use the *GIBBON* functions im2patch and patch2tri to get a watertight, i.e. closed and manifold, surface mesh.

For the alternative input form, namely skeletonized or synthetic graph networks, continuous surface models are obtained by using level-set images. The procedure for creating level-sets typically includes three steps. The initial step involves embedding the spatial data within an image domain. The second step entails computing a distance function between the spatial data nodes and the image’s voxel grid. The last step involves using the distance function to produce a (signed) level-set image. From there, continuous surface geometries can be obtained through the calculation of isosurfaces. A detailed description of this method can be found in [10] and an implementation of it already exists in *GIBBON*. Based on the graph network’s size and the desired level of precision, calculating the distance function between the network nodes and the image voxel grid can become significantly time-consuming. To address this, we enhance the efficiency of the function by creating a binary image from the graph network, enabling the distance map calculation to occur only between a dilated version of this binary image and the nodes of the graph network.

### 2.3. Surface mesh processing

The watertight surface mesh, outlined in the previous section, exhibits voxel artefacts. A remeshing step is therefore required to eliminate these artefacts. We use *Geogram* which is an external library in *GIBBON* to remesh the triangulation defined by the mesh faces (F) and the vertices (V) [42]. In particular, the code *Vorpalite* is used and optimizes the Voronoi diagram from the point of view of sampling regularity. The resulting mesh consists of a near-isotropic distribution of triangles that transforms the stair-shaped surface patterns to a smoother appearance. We follow that by using the patchSmooth function available in *GIBBON* to complete the smoothing operation. In particular, we use the option of ‘Humphrey’s Class’ that preserves best the overall volume of the watertight surface mesh.

However, after the smoothing step, small holes might appear in the surface mesh, leading to the loss of its watertightness. To repair it, we use PyMeshFix [65] from the PyVista library [66]. For compatibility purposes, we use the trimesh library [67] to read and write the defective and repaired mesh, respectively.

### 2.4. Skeletonization

In order to run physics-based simulations, it is necessary to assign boundary conditions on the edges of the network (for example to impose fluid pressure, velocity or solute concentration conditions). This requires creating flat surfaces at the terminal branches of the network and labelling them. While this task can be done manually for small networks, it becomes cumbersome and time consuming for medium and large networks. Thus, automating this process is crucial to make this pipeline viable for a broader spectrum of applications. One approach to automation involves baselining the cutting and labelling process around the terminal branches of a network’s centre-line volume. Here we describe the step in the pipeline which creates the centre-line for the tubular volumes.

We assume that the centre-line of the vessels is equivalent to the skeleton obtained by skeletonization algorithms in the context of image-processing. An easily accessible open-source tool for three-dimensional tubular-dataset analysis is therefore needed. Moreover, to facilitate automation, this tool must be designed to operate as a standalone executable application, negating the need for graphical user interface (GUI) interaction. VesselVio [43] meets these requirements and is compatible with both Windows and MacOS. It also provides high-level Python libraries, just-in-time compilation and parallel processing for rapid and detailed feature extraction techniques. This latest feature allows the transformation of the skeleton image to a graph format. For Linux users, the *TreeSkel* package [44], employing the minimum cost paths algorithm [68], offers an alternative solution for skeletonization.

The smoothed surface mesh was converted back into a binary image, due to the potential alterations introduced by the remeshing and smoothing operations on the original binary image. *VesselVio*, or alternatively *TreeSkel*, was then used to derive the skeleton of the network. Furthermore, plugins were created to import the graph format output into *MATLAB*. However, when *VesselVio* is used, overlaying the skeleton on the surface mesh reveals a noticeable ‘retraction’ effect, where the skeleton’s edge points do not extend fully to the surface mesh. This is expected since *VesselVio* employs a custom implementation of a widely used medial axis parallel thinning algorithm [69]. This retraction poses a challenge as the edge points are crucial for guiding the slicing and labelling of surfaces to create boundary conditions. To address this issue, a ray-tracing algorithm [70] is employed to elongate the edge sub-segments until they intersect with the surface mesh. These intersection points are then integrated into the skeleton, serving as the new edge points.

### 2.5. Anisotropic mesh to enhance computational accuracy and efficiency

Before using the skeleton edge points and terminal branches to guide the cutting and labelling of the surface mesh, it is essential to perform an important step, especially for networks composed of vessels with a wide range of diameters. In fact, users may encounter a challenge in selecting an appropriate element size for the isotropic mesh. Choosing a small element size better preserves the morphology of the smaller tubular structures, yet results in excessive meshing of the larger ones, leading to a significant increase in the computational time required for the physics-based simulations. On the other hand, opting for a larger element size can lead to inaccuracies in representing the morphology of smaller tubes, along with a deficiency in the number of elements on the edge surfaces where boundary conditions are applied. To address this issue, employing an anisotropic meshing approach, where the degree of anisotropy is guided by the tubes’ radii, is required. A minimum distance is thus calculated between the nodes of the surface mesh and the skeleton nodes using the minDist function found in *GIBBON*. This data is then fed to *TetGen* [41], an external library interfaced by *GIBBON*, which generates a volumetric mesh and from which we extract the anisotropic surface mesh. In this resulting mesh, smaller vessels are finely detailed, whereas larger vessels are meshed more coarsely.

It is important to note that the mesh refinement presented in this section is based solely on morphological considerations. However, depending on the underlying physics of the problem, further refinement may be required to adequately resolve element sizes, capture sharp gradients and minimize numerical errors—particularly in cases involving high Reynolds numbers and strong gradients at the interfaces.

### 2.6. Slicing and labelling to enable boundary condition assignment

To assign boundary conditions, it is essential to generate terminal cross-section surfaces through the tubular structure. For broader applicability, these surfaces should be flat, particularly to provide sufficient flexibility for use of vectorial entities, and they must contain a sufficient number of mesh elements to avoid numerical instabilities. This can be accomplished by slicing the surface mesh’s terminal branches along their edges and subsequently remeshing to ensure the entire mesh remains watertight. Thus, accurate slicing operations are required at each terminal branch.

A slicing plane is defined by a point and a normal vector. For each slicing operation performed on the tubular geometry, the point of origin is an edge point of the skeleton, and the normal vector corresponds to terminal sub-segment of the skeleton. However, since a slicing plane is infinite, it might indiscriminately affect other sections of the mesh. To enhance the accuracy of this operation, a Boolean constraint is introduced, ensuring that only interconnected elements nearest to the skeleton edge point are impacted. This is achieved by using the minDist function to apply the Boolean condition, followed by the triSurfSlice function for executing precise slicing on the targeted branch. Both functions are part of the *GIBBON* toolbox.

Following that, the now open surface mesh is re-sealed by remeshing the terminal branches using a series of functions, the most important of which is regionTriMesh3D (available in *GIBBON*). Each newly formed surface is assigned a distinct label, and an automatically generated text file, containing boundary conditions applied to those surfaces, is created. This text file can be directly communicated to *FEniCS* and is essential in the automation of the finite element simulations.

### 2.7. Volumetric meshing

The labelled surface mesh is now prepared for a second processing by *TetGen* [41] to create a volumetric mesh, ready for physics-based simulations. We input specific parameters into *TetGen*, including meshing options, faces, nodes, face labels and the number of regions. The runTetGen command in *GIBBON* is then used to produce a tetrahedral mesh. The output includes the tetrahedral elements, element IDs, faces, face IDs and the coordinates of the nodes. It is also possible to convert the tetrahedral elements to hexahedral ones using the tet2hex function. *GIBBON* also provides the capability to extrude the triangular surface elements into pentahedra, making the mesh suitable for applications like fluid–structure interaction.

### 2.8. Mesh exporting and conversion

Different finite element (FE) software packages require various mesh formats. The FE software used in our pipeline is *FEniCS*, which supports a limited number of formats, including .*xml* and .*xdmf*. To accommodate this, we have developed a function capable of converting the output from a *TetGen* mesh into a version 2 ASCII *Gmsh* file (.*msh* format). Subsequently, a Python script employing the *meshio* library is used to convert the .*msh* file into a .*xdmf* format, ensuring compatibility with *FEniCS*.

### 2.9. Mathematical models

We integrate physics-based mathematical models into this workflow, which are commonly utilized in simulating processes that involve (embedded) tubular objects. The first model is the incompressible Navier–Stokes model to simulate the fluid dynamics in the tubular objects. The second model is to simulate the advection–diffusion–reaction of specie(s) (solutes, oxygen, drugs, nanoparticles, etc.) in the embedded or embedding domain. While these are specific examples, the broad framework can be easily adapted to other physical models (e.g. solid mechanics).

#### 2.9.1. Computational fluid dynamics

We assume that the fluids of interest are non-compressible Newtonian fluids and so fluid flow can be described via the incompressible Navier–Stokes equations, written in the form


(2.1) $$\begin{array}{lcr} & \rho (\frac{\partial u}{\partial t}+u\cdot \nabla u)=\nabla \cdot \sigma (u,p)+f, & \end{array}$$

(2.2) $$\begin{array}{lcr} & \nabla \cdot u=0, & \end{array}$$

where *f* is a given force per unit of volume, **u** is the velocity of the fluid, *p* its pressure, ρ its density and σ(*u,p*) is the stress tensor. For Newtonian fluids, the stress tensor is given by


(2.3) σ(u,p)=2μϵ(u)−pI,

where **I** is the identity matrix and ε(**u**) is the strain-rate tensor,


(2.4) $$\begin{array}{lcr} & \varepsilon (u)=\frac{1}{2}(\nabla u+(\nabla u{)}^{T}). & \end{array}$$

The Navier–Stokes equations exhibit nonlinearity, transience and a non-trivial coupling between pressure and velocity. While coupled methods exist to solve the system of equations, it is more popular to decompose the problem into several more manageable equations. The initial splitting scheme was proposed by Chorin [71] and Témam [72]. This method was further refined by Goda [73], who introduced a velocity correction step, and his approach became widely recognized in the literature as the incremental pressure correction scheme (IPCS). Despite the existence of alternative splitting schemes, a thorough comparison presented in [74] indicates that, in terms of both efficiency and accuracy, the IPCS generally outperforms other methods. Moreover, the IPCS method is more straightforward to implement when contrasted with alternative splitting schemes. It consists of solving three equations:

— the tentative velocity step (a convection–diffusion–reaction equation),
— the pressure-correction step (a Poisson equation),
— the velocity update step (a projection).

We customize the implementation of this method provided in the *FEniCS* tutorial [75].

#### 2.9.2. Species transport in the embedded or embedding domains

We include general-purpose transport of chemical species, described using an advection–diffusion–reaction equation,


(2.5) $$\begin{array}{lcr} & \underset{\begin{array}{c} \text{temporal evolution} \end{array}}{\underbrace{\frac{\partial c}{\partial t}}}=\underset{\begin{array}{c} \text{Diffusion} \end{array}}{\underbrace{\nabla \cdot (D\nabla c)}}-\underset{\begin{array}{c} \text{Advection} \end{array}}{\underbrace{\nabla \cdot (uc)}}+\underset{\begin{array}{c} \text{Reaction/Source} \end{array}}{\underbrace{g(c)}}. & \end{array}$$

Here the temporal evolution of a chemical species of concentration c is linked to its spatial evolution through diffusion, advection by a vector field u and/or a reaction within the domain. It is worth noting that this equation is usually coupled (weakly) with the aformentioned Navier–Stokes through the velocity field u especially when the advection effects are important.

#### 2.9.3. Mixed-dimension models: 3D–1D coupling

Our framework is generic and suitable for flow systems with high, intermediate or low Reynolds numbers. However, there are numerous situations where it is practical to consider steady-state flows at low Reynolds numbers, such as blood flow in networks of small blood vessels and plant root systems. In these cases, it is reasonable to disregard changes in flow velocity and inertial effects. This is reflected mathematically by eliminating both the time-dependent and inertial terms in the Navier–Stokes equations, leading to the use of the simpler Stokes flow equations. Moreover, in such situations, a one-dimensional model of the tubular structure is often adequate to capture the essential aspects of flow physics and is often used in the literature [14,15,17]. Nevertheless, when the focus is on how the one-dimensional tubular objects interact with their three-dimensional surrounding environment, a significant challenge emerges due to the mixed-dimensional (3D−1D) nature of the problem. In fact, various numerical methods vary in their approach to bridging the dimensional incompatibility between network and embedding domain by distributing source terms. The contribution of the source term within the encompassing bulk medium can be represented through line source terms [76,77], surface source terms [78,79] or volume source terms [4,18]. In many of these methods, it is assumed that the diameters of the network branches are significantly smaller than the dimensions of the surrounding domain. This facilitates a simpler mesh creation process that results in an overestimation of the bulk volume by covering the space occupied by the tubular structures. Additionally, due to the elimination of their physical structure, the tubes no longer offer a resistance to flow in the embedding domain. Finally, as these mixed-dimension models typically represent the network by a series of finite cylinders, inaccuracies arise at junctions where source terms should be distributed.

Most of these problems in 3D–1D coupling settings can be avoided if the exact network surface mesh is known. Our framework allows for the building of such continuous surface meshes from networks of one-dimensional segments, as shown in §2.2. Consequently, simulations conducted on the network of one-dimensional segments can be projected onto the network surface mesh using an minimal distance-based approach between the nodes of the one-dimensional segments and those of the surface mesh. Then, using the *TetGen* [41] interface with *GIBBON*, a conforming mesh that integrates the embedded system and its surrounding environment can be created. The interface conformity between the two domains allows the direct use of the distributed source terms on the network surface without the necessity of extrapolation.

### 2.10. Scientific visualization and post-processing

Geometries and meshes are visualized in *MATLAB* using the *GIBBON* open-source toolbox, whereas the simulation results are exported in .*vtk* format and are visualized and post-processed using *ParaView* [60,61] (built on the VTK library [62]). For automation purposes, the use of the *ParaView* GUI must be avoided. For that, generalized macros were written and called from *MATLAB* through a Command-Line Interface (CLI).

## 3. Results and discussion

This section highlights five case studies that comprehensively illustrate the capabilities of the Tube2FEM framework. Particularly, we present instances of tubular geometries corresponding to three distinct applications (lung airways, root–soil systems, micro/macro circulation), each emphasizing various aspects of the framework, including surface mesh reconstruction, meshing, CFD simulations, 3D−1D approaches, visualization, etc. We note that all the case studies presented in the upcoming section are automated, with each having all of its commands centralized within a single *MATLAB* script. Table 1 summarizes all the key user-defined parameters, method specifications and some of the output results for all of the five case studies. It is also worth noting that all computations were carried out on a personal laptop with the following specifications: 11th Gen Intel(R) Core(TM) i7-1185G7 processor @ 3.00 GHz, 1805 Mhz, four cores, eight logical processors and 32 GB of RAM. Finally, with the exception of the cases featuring patient-specific data, all the codes are made available on GitHub at: https://github.com/CheikhSleiman/Tube2FEM.

**Table 1.** Summary of key parameters, method specifications and performance metrics of Tube2FEM for the five case studies presented in this work.

			case study 1: lung airways	case study 2: root–soil	case study 3: microcirculation	case study 4: 3D–1D	case study 5: portal vein CFD
surface mesh processing	input		patient-specific three-dimensional segmented image	three-dimensional segmented image [80]	skeletonized network [81]	skeletonized network [81]	patient-specific three-dimensional segmented image
	reconstruction method		marching cubes	marching cubes	level-set	level-set	im2patch/patch2tri
	remeshing		anisotropic	isotropic	isotropic	isotropic	isotropic
	remeshing parameters	number of points	180k	100k	16.5k	16.5k	140k
	smoothing		yes	yes	yes	yes	yes
	smoothing parameters	smoothing method	Humphreys-Class smoothing (HC)	HC	HC	HC	HC
		number of steps	4	2	15	15	2
skeletonization	software		*VesselVio/TreeSkel*	—	—	—	*VesselVio*
	parameters	resolution	10	—	—	—	1
		prune length	7	—	—	—	7
		filter length	20	—	—	—	10
automated boundary conditions	slicing and labelling		no	no	yes	no	yes
	number of labelled surfaces		1	7	18	7	47
mesh generation and conversion	number of nodes		—	330k	32.9k	46.3k	865k
	number of tetrahedra		—	2M	149k	241k	5M
	number of domains		1	2	1	1	1
finite element	element type		—	—	—	P1	P2–P1/P1–P1
	boundary conditions		—	—	—	distributed surface sources *f* (*x*, *y*, *z*)	**Navier–Stokes:** inlet velocity = *f* (*t*) outlets pressure = 0 **Adv–Diff–Reac:** volumetric source: *f* (*x*, *y*, *z*)
	simulated time and time step		—	—	—	—	*T* = 1 s dt = 0.01 s
postprocessing and visualization	macro scripting		—	—	—	no	yes
computational time	surface mesh reconstruction and processing		1 min 52 s	25 s	4 min 20 s	4 min 20 s	2 min 15 s
	skeletonization		23 s	—	—	—	27 s
	automated boundary conditions		—	—	5 s	—	44 s
	mesh generation and conversion		—	45 s	10 s	19 s	3 min 22 s
	Finite element		—	—	—	5 min	NS: 1 h 8 min/Adv–Diff–Reac: 26 min
	Total		2 min 15 s	1 min 10 s	4 min 35 s	9 min 39 s	1 h 40 min

### 3.1. Case study 1: radius-dependent anisotropic mesh generation of lung airways

**Figure 2.**
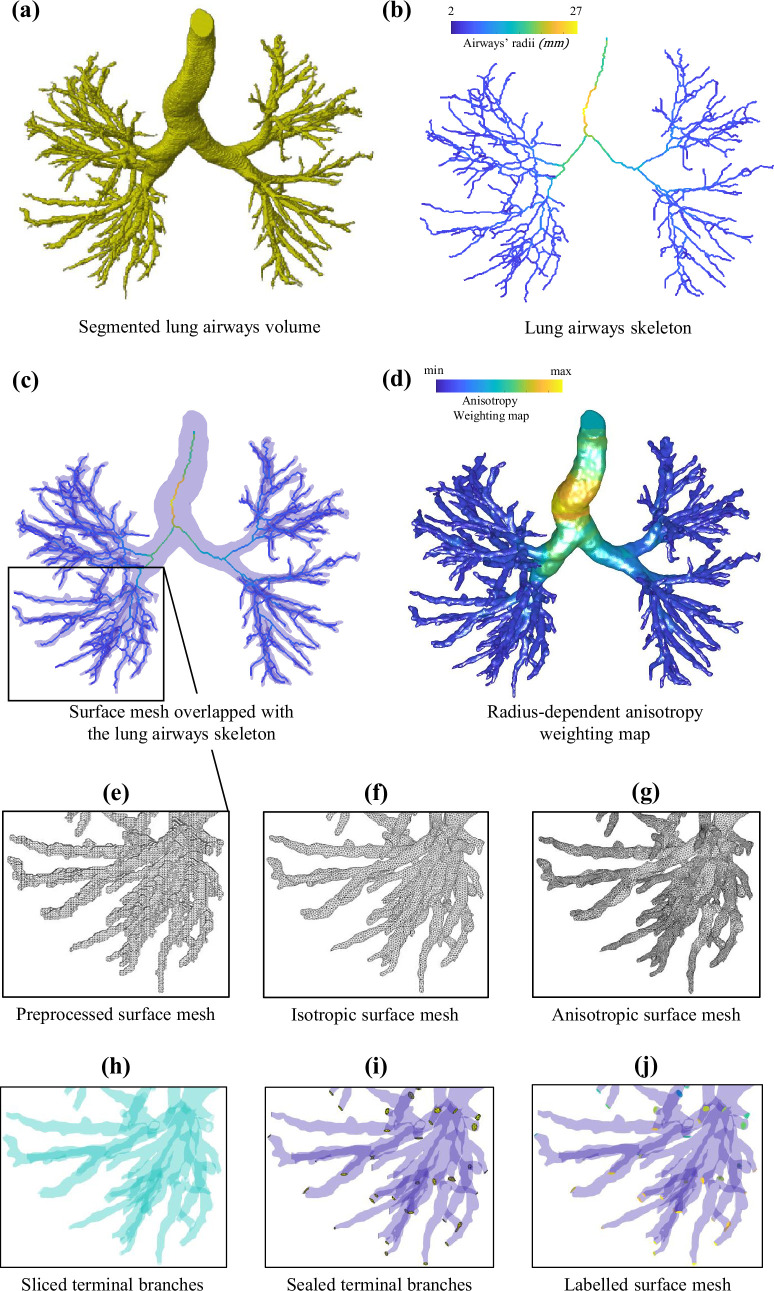
The Tube2FEM procedure for creating simulation-ready radius-informed anisotropic meshes for a lung airway tubular geometry acquired using medical CT imaging.

For our initial example, we employ Tube2FEM to generate simulation-ready meshes from segmented lung airways acquired using medical CT scans. The primary difficulty with this type of tree-like tubular geometry is its topological complexity, since it contains more than 10 branching generations, and there is significant variation in diameter between the smallest and largest airways. Indeed, the challenge stems from choosing a mesh element size that maintains the airways’ morphology without leading to increased computational time for the simulations. Tube2FEM addresses this issue by employing a radius-dependent anisotropic meshing approach. The process begins with the segmented lung airways volume, as depicted in figure 2a, from which the tubular structure’s centre-line is determined using skeletonization algorithms, as detailed in §2.4. Additionally, the surface mesh is generated from the three-dimensional image using the marching cubes algorithm or similar techniques, as described in §2.2. However, this mesh initially exhibits voxel artefacts, necessitating further processing steps such as remeshing and smoothing, discussed in §2.3. Subsequently, the processed surface mesh is overlaid with the skeleton, as shown in figure 2c. The next phase involves projecting the centre-line radii field onto the surface mesh, based on the minimal distance criterion outlined in §2.5. This projection creates a radius-dependent weighting map that guides the mesh’s anisotropy, shown in figure 2d. The final comparison between the preprocessed surface mesh, the isotropically remeshed and smoothed version, and the anisotropic mesh is presented in figure 2e–g respectively. Finally, figure 2h–j respectively illustrates the clipped terminal branches, sealed outlets and labelled boundary regions, demonstrating the readiness of the mesh for numerical simulation.

### 3.2. Case study 2: multi-domain mesh generation of a root–soil system

**Figure 3.**
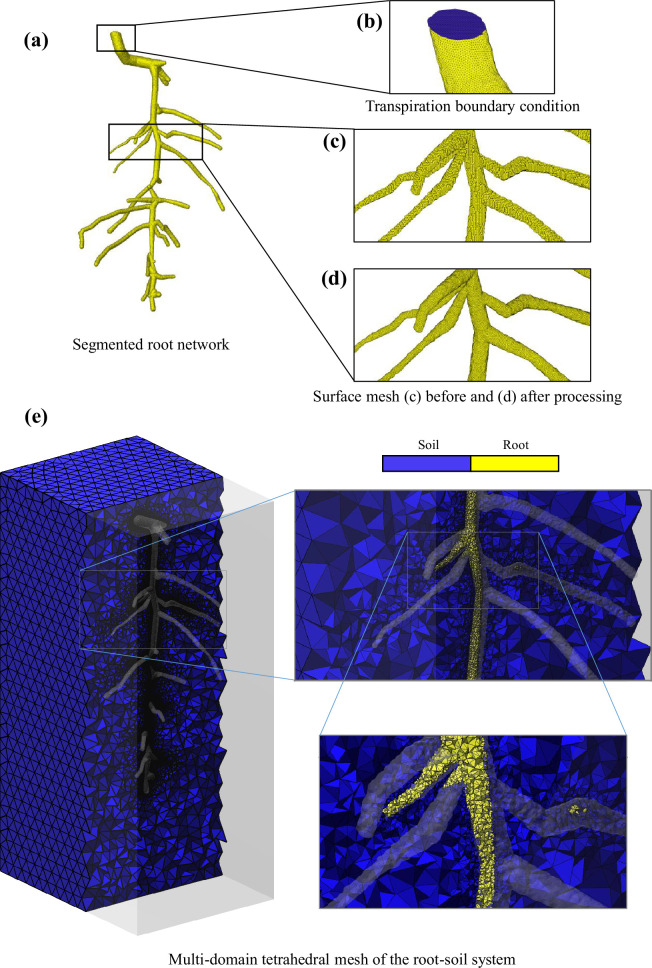
The Tube2FEM procedure for creating a multi-domain tetrahedral mesh of a root–soil system, based on CT data.

In this case study, Tube2FEM is used to generate a multi-domain, simulation-ready mesh of a root network embedded within a soil medium. The segmented root network data, previously examined in [4], was accessible online only in *Gmsh* (.*msh* format) as referenced in [80]. Since the root–soil system was originally imaged through X-ray tomography before being segmented and processed into a meshing format, we converted the dataset back into a standard imaging format (.*tiff*) and reprocessed it from the beginning using our framework. We then use this segmented (.*tiff*) dataset illustrated in figure 3a as the input for our procedure. As in the previous case study, we extract the surface mesh using the marching cubes algorithm or equivalent techniques, as outlined in §2.2. Remeshing and smoothing processes are then carried out to eliminate voxel artefacts and achieve a desired mesh density, as illustrated in figure 3c,d. Indeed, in order to produce reliable results in hydraulic root–soil simulations, the mesh needs to be carefully locally refined at the interface. This is particularly important as drying soil around the roots creates large and highly localized pressure gradients. The effective way to capture these gradients is through local mesh refinement at the interface [4]. To represent the plant’s transpiration activity above the soil, a designated surface was labelled to impose a transpiration flux boundary condition, as illustrated in figure 3b. A soil medium is then built around the root mesh using *GIBBON*’s triBox function, and both media are inputted into *TetGen*, as detailed in §2.7, to create a multi-domain tetrahedral mesh. Figure 3e shows both domains, featuring a mesh density gradient from the interface to the boundaries of the soil domain.

### 3.3. Case study 3: generating surface and volumetric meshes of a microcirculation blood network

This case study demonstrates the ability of Tube2FEM to generate simulation-ready labelled surface and volumetric meshes from networks of one-dimensional tubular objects. These networks can either be synthetic (for example, via generative algorithms) or result from skeletonization processes applied to imaging datasets. While the procedure is general, we apply it to a widely adopted microvascular network dataset used in the literature to explore microcirculatory blood flow and transport processes, studied initially in [81].

**Figure 4.**
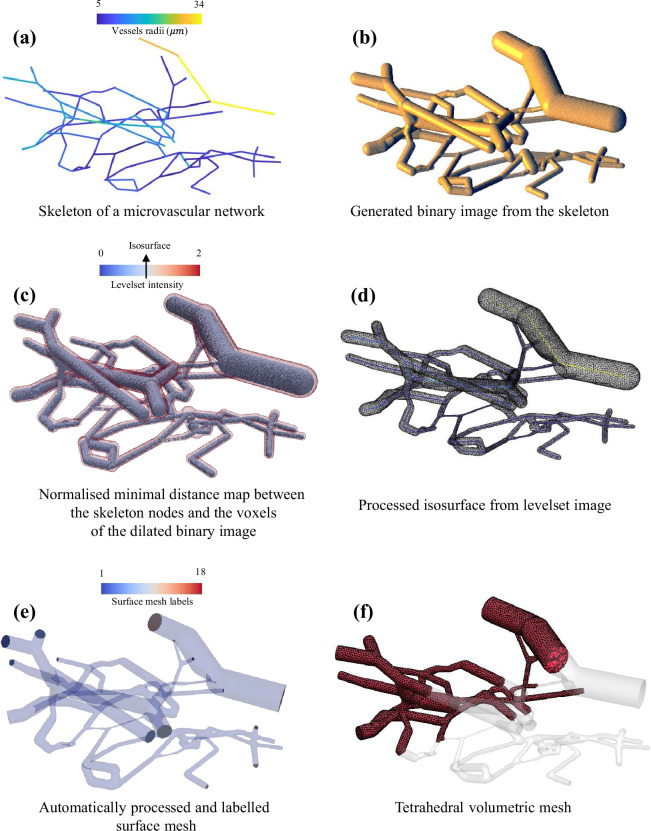
The Tube2FEM procedure for generating labelled surface and volumetric meshes from a skeletonized microcirculation tubular geometry [81].

The level-set method, detailed in §2.2, is used here to create a watertight surface mesh. The starting point is the vascular network centre-line over which the vessel radii field is presented, as illustrated in figure 4a. The second step entails embedding the spatial graph within an image domain and then computing a distance function between the centre-line nodes and the image’s voxel grid. This method can become significantly time consuming if the image domain is not carefully chosen. To address this, we create a binary image from the graph network as illustrated in figure 4b. Several image dilation operations are then executed to establish the required image domain. The signed level-set image, shown is figure 4c can be interpreted as the normalized minimal distance map between the centre-line nodes and the dilated binary image. The isosurface, corresponding to level-set intensity = 1, can be extracted from the level-set image. The resulting surface mesh, which has been remeshed and smoothed to eliminate artefacts, is displayed in figure 4d. The following step involves applying the slicing and labelling algorithm, described in §2.5, to assign boundary conditions. This is achieved by overlaying the centre-line network with the processed surface mesh and identifying the edge nodes and the directionality of the edge segments. Finally, having the watertight labelled surface mesh illustrated in figure 4e, it is possible now to generate the tetrahedral mesh using the *TetGen* interface in *GIBBON*. The volumetric mesh in shown in figure 4f.

### 3.4. Case study 4: 3D–1D coupling strategy to simulate tissue perfusion

This case study aims to demonstrate the ease of 3D−1D coupling between an embedding tubular network and its surrounding domain using Tube2FEM. For convenience, we apply this procedure on the same graph network used in case study 3 and the same reconstructed surface mesh which was created using the level-set method. The final goal is to simulate the extra-vascular solute transport driven by a heterogeneous distribution of solutes within the vascular network.

**Figure 5.**
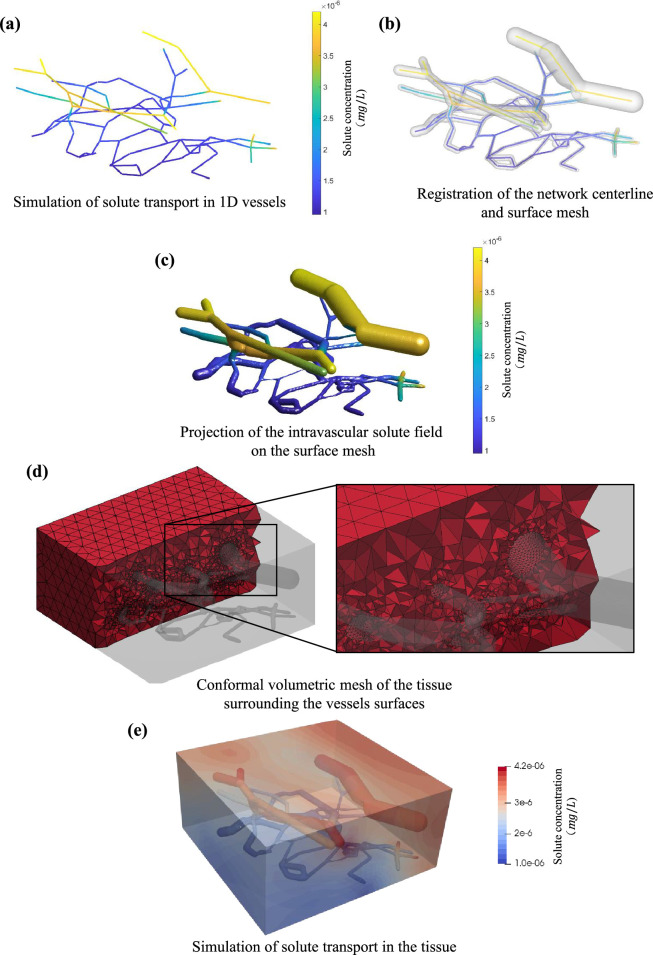
A 3D−1D coupling strategy enabling solute transport from a microcirculation vascular network composed of one-dimensional segments into the surrounding three-dimensional biological tissue [81].

In this specific scenario, a one-dimensional representation of each vessel suffices to capture the flow physics. This renders the computational time cost of the simulated intravascular processes very cheap in comparison with a three-dimensional case scenario. Consequently, the initial step in this process involves simulating solute transport across a network of one-dimensional vessels. This simulation can be conducted using methods such as finite element, finite volume among others [14,15]. However, when attention shifts to the interactions between the one-dimensional tubular objects and their three-dimensional surroundings, a considerable challenge arises from the mixed-dimensional (3D−1D) nature of the boundary. As discussed in §2.9, different numerical methods adopt various strategies to overcome the dimensional mismatch between the network and the embedding domain by distributing source terms (line, surface or volume sources terms). For this case study, we distribute the source terms across the surface, since Tube2FEM is capable of reconstructing the exact surface mesh of the vascular network, as demonstrated in case study 3. To achieve this, we proceed to overlap the simulation results on the centre-line with the reconstructed surface mesh as illustrated in figure 5b. Then the solute field on the network of one-dimensional vessels is projected on the mesh using a minimal distance computation between the centre-line nodes and the mesh ones. This results in the surface field shown in figure 5c. Following this, we create the surrounding tissue volumetric mesh using *TetGen–GIBBON* interface. In the input options for *TetGen*, we ensure that the volumetric tissue mesh should be hollowed out from the vascular domain, which is represented by the reconstructed surface mesh. We also ensure that the mesh density of the vascular surface mesh is sufficiently fine to more effectively resolve the volumetric mesh density gradient near the vascular–tissue interface. The result of these operations can be visualized in figure 5d. Additionally, we note that the internal surface of the volumetric mesh, created by the removal of the vascular domain, conforms to the reconstructed vascular surface mesh. Such conformity is essential for facilitating consistent interactions between the different domains. The volumetric mesh produced is first exported in the (.*msh*) format and subsequently converted to the (.*xdmf*) format using the *meshio* package. This latter format ensures compatibility with the *FEniCS* software, which is employed to carry out the finite element analysis. We also export the solute field evaluated on the vascular surface mesh in a tabular format ($x_{N}$, $y_{N}$,$z_{N}$,$c_{s}$), where $x_{N}$, $y_{N}$ and $z_{N}$ are the vascular surface mesh nodes coordinates and $c_{s}$ is the concentration of the solute on the node N. This table is then communicated to *FEniCS* and is used in assigning boundary conditions. Finally, we use the Poisson equation to model a simple steady-state solute diffusion process in the tissue as illustrated in figure 5e. We note that this procedure can be used for much more complex problems, and can be further developed, for instance to account for time-dependent intravascular flow variations and two-way coupling.

### 3.5. Case study 5: detailed computed-tomography-to-simulation procedure for tubular geometries: application to portal vein network

**Figure 6.**
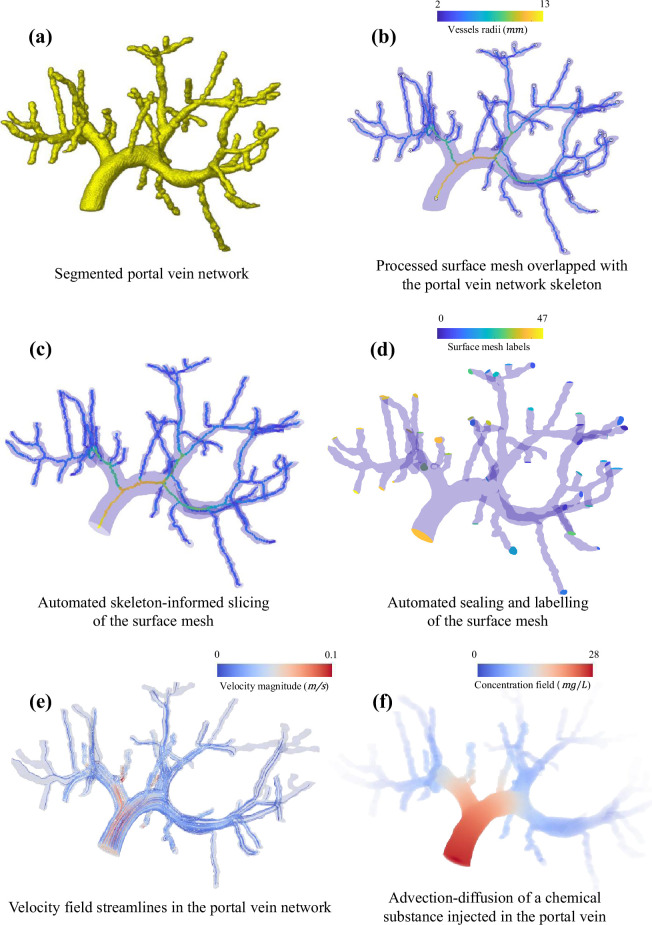
Image-based procedure detailing all the necessary steps to conduct CFD and advection–diffusion–reaction simulations from a segmented vascular geometry.

This final case study is designed to showcase a comprehensive framework for conducting physics-based simulations on tubular geometries obtained via imaging. While the framework is broadly applicable, we demonstrate its use through a portal vein network derived from a patient’s CT scan. This segmented network is illustrated in figure 6. Similarly to previous case studies, the portal vein surface mesh can be extracted using the marching cubes algorithm or an equivalent method detailed in §2.2. The surface is then remeshed to a desired density and smoothed to get rid of geometrical artefacts. Following that, we call *VesselVio* software in *MATLAB* and skeletonize the binary image. The overlap of the resulting centre-line and the surface mesh can be seen in figure 6b. Subsequently, the skeleton edge nodes are identified, and when combined with the direction of the skeleton terminal sub-segments, they determine the slicing planes. The surface mesh after the slicing operations is visualized in figure 6c. The open geometry is then sealed by remeshing and labelling the open surfaces near the skeleton terminal branches. The detailed slicing, sealing and labelling procedures are detailed in §2.6. In total, figure 6d shows 47 labelled surfaces, 46 of which are created by sealing the sliced geometry and one being the vessel’s wall. Those labelled surfaces are crucial to assign boundary conditions for the physics-based models. For the Navier–Stokes (NS) simulation, we apply a time-dependent velocity to the inlet, zero pressure to the outlets and zero velocity to the vessel walls (no-slip boundary condition). Figure 6e illustrates the velocity streamlines at a specific time point, resulting from solving the NS problem. We then save all the velocity fields at the different time steps and use them to inform the advection of the chemical specie in the advection–diffusion–reaction problem. Figure 6f shows the resulting concentration of the chemical field within the portal vein network. It is important to highlight that those simulation results are demonstrative of the capacity of the pipeline to solve complex flow and flow-related problem. The set of parameters and associated boundary conditions used in those simulations do not necessarily represent patient-specific physiological ranges.

## 4. Perspective

Anticipating future advancements of our pipeline, we highlight critical improvements targeted at enhancing the accuracy and reducing the computational time required for simulating (embedded) tubular structures. The outlined perspectives below may be explored in future work to enhance the framework’s efficiency and technical capabilities.

— *Use of high-performance finite element Navier–Stokes solver*. Oasis is a Python-based high-performance finite element Navier–Stokes solver [82]. It utilizes foundational components from the *FEniCS* project and is designed for addressing large-scale applications involving intricate geometries, particularly on massively parallel computing clusters.
— *Achieve faster periodic convergence in case of cyclic loading*. The computational expense of three-dimensional biomedical fluid dynamics simulations is often attributed to the need for computing multiple cycles (cardiac, respiratory, etc.) before achieving a periodic solution. The work of Pfaller *et al.* [83] showed the possibility of fastening the periodic convergence by generating appropriate initial conditions using the simulation results of reduced-order one-dimensional models.
— *Automating a comprehensive verification of CFD simulation quality*. A few decades ago, multiple fluid mechanics editorial policy boards stated that ‘there is a need for higher standards on the control of numerical accuracy’ and that ‘a single calculation of a fixed grid is not acceptable’ [84]. However, recent research indicates that even grid refinement is insufficient for evaluating simulation quality. Instead, a comprehensive CFD investigation involving solver numerics, mesh and time-step refinement is essential. For instance, it has been shown in [85] that robust and minimally dissipative CFD solvers can tolerate surprisingly coarse resolutions, whereas solvers using low-order and/or stabilization schemes may require much higher resolutions to detect relevant flow patterns.
— *Include lumped parameter network (LPN) models for boundary conditions*. While ensuring an accurate geometric representation of the computational domain is paramount in simulating fluid flow, it is equally essential to emphasize the importance of realistic boundary conditions. In general, the distribution of flow and pressure field within the simulated domain is often unknown and challenging to specify at the inflow/outflow boundaries. In fact, the physiology of the flow at the inlet/outlet is often too complex to be represented by a fixed-value Dirichlet of Neumann-type boundary condition. An alternative strategy involves linking the solution at the inflow/outflow boundaries of the modelled domain with simplified lumped parameter (zero-dimensional models) or one-dimensional models representing the missing domain [86,87]. This often requires solving an ODE to describe the physics of the flow at the boundary condition level.
— *Implementing two-way coupling for embedded systems*. Regarding the embedded systems, it is necessary to establish a two-way coupling between the embedded and the embedding domain. This coupling is necessary to ensure the conservation of mass (e.g. for mass transfer problems in vascular/tissue, root–soil and well–soil systems), momentum (e.g. to account for mechanical deformations in expanding or contracting airway/lung systems) and energy (e.g. for heat transfer problem in heat exchangers).

## 5. Conclusion

In this paper, we developed Tube2FEM, an open-source highly automated framework for flow and flow-related processes simulation in (embedded) tubular structures. This system is capable of handling various input forms, including segmented tomographies and synthetic or skeletonized networks. It offers a range of features that include creating surface models, automated slicing and labelling for the assignment of boundary conditions. Furthermore, it supports surface and volumetric mesh generation and allows performing simulations using the FEM. It also offers capabilities for post-processing and visualizing the results. The framework utilizes a selection of carefully curated open-source softwares and libraries, chosen to guarantee a high degree of versatility for various simulation scenarios. Among the most significant of these are *GIBBON*, which is essentially employed for image and/or geometry processing, *FEniCS* for conducting FE simulations, and *Paraview*, which is utilized for the post-processing tasks.

The case studies presented in this work demonstrate the comprehensive capabilities of the Tube2FEM framework across a range of applications of (embedded) tubular geometries, from lung airways and root–soil interactions to macro/microcirculation and tissue perfusion. We showcase in all case studies advanced meshing capabilities including surface mesh creation and extraction, isotropic/anisotropic remeshing, smoothing, multi-domains and labelling for automated boundary conditions. Additionally, we showcase in case studies 4 and 5 a range of physics-based models applied to tubular and embedded tubular systems. Particularly, a framework for 3D−1D mixed dimensions simulation for embedded tubular system was created and was used to simulate the solute transport from a microcirculation network to its surrounding biological tissue. In addition, a CT-to-simulation framework was also developed to conduct CFD and advection–diffusion–reaction simulations on image-based geometries.

Finally, future work will focus on enhancing the automation and efficiency of the workflow, expanding the framework’s capability to handle complex boundary conditions and couplings, and on integrating high-performance solvers to achieve faster computational time while maintaining the results precision.

## Data Availability

Data and relevant code for this research work are stored in GitHub: [88] and have been archived within the Zenodo repository: [89].
